# Teaching computational genomics and bioinformatics on a high performance computing cluster—a primer

**DOI:** 10.1093/biomethods/bpac032

**Published:** 2022-11-15

**Authors:** Arun Sethuraman

**Affiliations:** Department of Biology, San Diego State University, 5500 Campanile Drive, San Diego, CA 92182, USA

**Keywords:** HPC, genomics, bioinformatics, curriculum

## Abstract

The burgeoning field of genomics as applied to personalized medicine, epidemiology, conservation, agriculture, forensics, drug development, and other fields comes with large computational and bioinformatics costs, which are often inaccessible to student trainees in classroom settings at universities. However, with increased availability of resources such as NSF XSEDE, Google Cloud, Amazon AWS, and other high-performance computing (HPC) clouds and clusters for educational purposes, a growing community of academicians are working on teaching the utility of HPC resources in genomics and big data analyses. Here, I describe the successful implementation of a semester-long (16 week) upper division undergraduate/graduate level course in Computational Genomics and Bioinformatics taught at San Diego State University in Spring 2022. Students were trained in the theory, algorithms and hands-on applications of genomic data quality control, assembly, annotation, multiple sequence alignment, variant calling, phylogenomic analyses, population genomics, genome-wide association studies, and differential gene expression analyses using RNAseq data on their own dedicated 6-CPU NSF XSEDE Jetstream virtual machines. All lesson plans, activities, examinations, tutorials, code, lectures, and notes are publicly available at https://github.com/arunsethuraman/biomi609spring2022.

Key messages

- Here, I describe the curriculum and logistics of implementing a semester-long course in computational genomics and bioinformatics on a high-performance computing cluster.
- Topics covered included genome assembly, alignment, phylogenomics, GWAS, RNAseq analyses, and population genomics.
- All curricular materials are available for other educators on the author’s GitHub page.

## Introduction

Studies that apply genomics, that is, that incorporate the entirety of heritable DNA from individuals, cells, strains of the same or many species have super-exponentially grown in the last decade [1, 2]. While this growth can be partially attributed to the seemingly ever-declining costs of DNA-sequencing on high-throughput platforms such as Illumina [copyright], Pacific Biosciences [copyright], IonTorrent [copyright], and Oxford Nanopore Technologies [copyright], the associated costs and need for storing, analyzing, and making sense of large genomic data have grown significantly (https://www.genome.gov/2020SV). Correspondingly, the market for bioinformatics and genomics skills has grown across the world, with multi-billion USD market sizes [3].

This puts the onus on university-level education on training current students on effective handling, analyses and interpretation of genomic data [4], as much as on accessible high-performance computing (HPC) facilities. However, college-level training on bioinformatics and genomics is lagging significantly on the applications of current tools and software pipelines for genome-size data analyses owing to (1) lack of accessibility to HPC facilities locally for classroom needs, (2) current reliance on students and trainees to install and run large genomic data analyses on their own laptops or devices, which can often overwhelm and crash current architecture, (3) lack of cross-compatibility of tools between operating systems and architectures, and (4) reliance on unsecured and slow network capabilities for web-based analyses and transfer of data [5].

To address all these issues, I developed and taught a comprehensive course on computational genomics and bioinformatics at San Diego State University in Spring 2022. The course was co-listed as part of the Computer Science, Biology, and Bioinformatics curricula, and the student trainees comprised graduate and senior undergraduate students at San Diego State University (SDSU) with varied research and training interests. Student registrants were either required to have some basic computer science background, with prior coursework in either databases (e.g. CS 503 Scientific Database Techniques or CS 514 Database Theory and Implementation), or programming (e.g. CS 200 Introduction to Data Science and Python), or enrollment in a graduate program in bioinformatics or biology. The students were not required to be enrolled in active research credits, although a majority of those enrolled were performing lab work with faculty across the Department of Biology. Considering the heterogeneous expertise of students, this course was developed to therefore encompass theoretical, statistical, algorithmic, and applied aspects of genomics (Table 1), that included analyses and quality control of raw genomic data, *de novo* assembly, read alignment to reference genomes, variant calling, phylogenomic reconstruction, *ab initio-* and reference-based annotation, population genomic analyses, genome-wide association studies (GWAS), and differential gene expression using RNAseq data. Lab sessions were developed based on analyses of publicly available genomic data from various sources including SARS-CoV-2 genomes from GISAID and NCBI, population genomic and phenotypic datasets from DataDryad and Zenodo, human genomic data from the 1,000 Genomes Project, and RNAseq data from EBI. All laboratories were designed to run on personalized 6-CPU virtual machines created for each student on NSF XSEDE [6] Jetstream (Indiana University) which provides a convenient web-based interface for large-scale data analyses to run via a web-browser. This, combined with the convenience of hybrid (in-person in a classroom equipped with computers and virtual) instruction via videoconferencing permitted accessibility of cutting-edge training in genomics for all students in the midst of the COVID-19 pandemic. Students were trained in Unix shell scripting and statistical analyses and data visualization in the R programming language as part of the course, but were provided the freedom to use a programming language of their choice and expertise for all assignments, laboratories, and examinations. I hope that this primer describing the course will serve as a helpful model for the genomics training community in developing similar courses at their own institutions.

**Table 1:** Schedule of lectures and associated laboratories from the Spring 2022 offering of BIOMI 609 Computational Genomics and Bioinformatics at San Diego State University

Lecture	Laboratory
Introduction to genomic sequencing technologies, datatypes	
Base calling, quality control	
Genome assembly—*de novo*, guided	Laboratory 1—genome assembly
Review + midterm 1	
Alignment, BLAST, Burrow–Wheeler transforms	
Variant calling	Laboratory 2—variant calling
Phylogenomics (neighbor joining, parsimony)	
Phylogenomics (likelihood)	Laboratory 3—Phylogenomics
Review + midterm 2	
Population genomics—1 (HWE, LD)	
Population genomics—2 (structure, evolutionary history)	Laboratory 4—Population genomics
Human genomics—GWAS	Laboratory 5—GWAS
mRNAseq analyses	Laboratory 6—RNAseq analyses
Annotation—*ab initio*, hidden Markov models	Laboratory 7—Genome annotation
Final examination review	
Final examination	

*Notes*: All laboratory manuals are accessible at https://github.com/arunsethuraman/biomi609spring2022/tree/main/Labs.

## Course organization and logistics

A request for funding/computational startup was made through the XSEDE education allocation portal (https://allocations.access-ci.org/) in January 2022, prior to commencement of the Spring 2022 term for 500,000 CPU hours to be shared by students of my BIOMI 609 Computational Genomics and Bioinformatics course. XSEDE (now completely transitioned into ACCESS—https://access-ci.org/) offers free educational allocations for most US-based educators and researchers, and has served as an invaluable resource for equitable computational use. My resource allocation application was reviewed and awarded seamlessly, with student accounts created easily through my computational startup account portal. Followed by this, students were required to create accounts on the XSEDE portal, with dual-factor authentication, which permitted the creation of individual virtual machines for each student. For convenience of using several preinstalled libraries and tools for genomics, every student thereon created a “Genomics Toolkit v.1.3.1” instance on their allocation, which is a precompiled virtual machine with 6 CPUs, 16 GB of RAM, and 60 GB of drive space, and pre-installed tools including bedtools [7], bcftools [8], and the bioconductor package [9]. A full list of tools on the Virtual Machine (VM) are available here https://iujetstream.atlassian.net/wiki/spaces/JWT/pages/334397443/Genomics+Toolkit+image. Additional tools that were required for each laboratory were installed individually by students on their VMs. The convenience of the Jetstream web interface allowed students a GUI for their Unix virtual machines through XSEDE, as well as easy file transfer to and from their local machines via a web browser. This permitted students with different degrees of expertise in Unix operating systems to learn and master the system via terminal and a web browser with minimal difficulty. Students were provided a detailed manual explaining how to navigate their Jetstream machines including file access, downloading and installing tools, setting paths, creating/copying/moving directories, writing and executing shell scripts, and exploring and summarizing large genomic datasets, prior to lab sessions. The course was organized such that students were introduced to a topic through lectures for a week or two, followed by which they spent a lab session working through analyses of real genomic datasets. Student performance was assessed through regularly interspersed graded analysis/programming assignments, take-home examinations, and a final project report (all materials accessible via the course’s GitHub page). All lectures and notes were also recorded and made accessible to the students immediately after every class. All videos of lectures and laboratories are accessible via this YouTube playlist: https://youtube.com/playlist?list=PL1e4GDlV5mnTQPoB7HR8UxQpq5stdMLiO. All lecture notes are accessible via the course’s GitHub page: www.github.com/arunsethuraman/biomi609spring2022. While my course was deployed onto NSF XSEDE, resources for this curriculum should be straightforward to extend and deploy onto other HPC clusters or cloud instances, such as Google Cloud (https://cloud.google.com/edu/faculty), AWS Educate (https://aws.amazon.com/education/awseducate/), the EU’s CoE (https://www.hpccoe.eu/eu-hpc-centres-of-excellence2/), or other similar education initiatives the world over.

## Laboratories

### Genome assembly

The first laboratory of the semester introduced students to downloading and processing genomic data from the Sequence Read Archive (SRA) using SRAToolKit v.3.0.0 (https://hpc.nih.gov/apps/sratoolkit.html), read quality assessment using fastqc v.0.11.9 (https://www.bioinformatics.babraham.ac.uk/projects/fastqc/), trimming of reads based on quality using trimmomatic v.4.0 [10], genome assembly from raw reads using the short read assembler, velvet v.1.1.0 [11], and assembly quality assessment using QUAST v.5.1 [12]. Students worked with SARS-CoV-2 genomes downloaded from the SRA, following which they were tasked with (1) writing their own program for visualizing the PHRED quality score distribution from a given FASTQ file and (2) assembling the same genome using several *de novo* methods such as velvet [11], AbySS [13], SOAPdeNovo2 [14], and SPaDes [15] and assessing assembly quality against the SARS-CoV-2 reference genome (NCBI Accession ID: NC045512.2) using QUAST [12]. This exercise introduced students to several genomic concepts, including quality scores, N50, N90, contiguity, and GC content, apart from setting the stage for accessing, downloading, processing, and visualizing large genomic datasets.

### Variant calling and phylogenomic reconstruction

In Laboratories 2 and 3, students were introduced to a variant calling pipeline using publicly available whole SARS-CoV-2 genomes from GISAID (https://www.gisaid.org/). Students utilized the GISAID data portal to search for specific strains, sorted on geographical origins to obtain FASTA files of whole assembled genomes, followed by alignment using clustalw2 [16], indexing using faidx [8], alignment to a reference genome using bwa-mem2 (https://github.com/bwa-mem2/bwa-mem2) [17], and variant calling using SAMtools and BCFtools [8] to create indexed variant call format (VCF) files. Additionally, whole-genome alignments were constructed using MAFFT v.7.90 [18] and whole-genome phylogenies constructed using RAxML-NG v.1.1.0 [19, 20], and visualized using FigTree v.1.4.4 (https://github.com/rambaut/figtree/releases). Associated concepts including computing genotype likelihoods, inferring molecular evolutionary history under the nearly neutral theory, the molecular clock hypothesis, and building phylogenies using distance-based and likelihood-based methods were discussed in detail during lectures. Additionally, the intuitive visualizations of the evolution of SARS-CoV-2 and other viruses offered by www.nextstrain.org provided an exciting and timely learning tool for the extensive utility of real-time genomics and strain and spread monitoring.

### Population genomics

In lectures leading up to the laboratory, students were introduced to several population genomics concepts, including a review of Mendelian inheritance; the Hardy–Weinberg principle; linkage disequilibrium and null expectations for an equilibrium population, genetic drift, and the Wright–Fisher process, basic coalescent theory (including expectations of time to coalescence, tree shapes, and the site frequency spectrum), quantifying and testing for natural selection using summary statistics, selective sweeps, the Wahlund effect and population structure, inbreeding, migration, and mutational processes. In the following laboratory session, students were led through estimation of haploid nucleotide diversity, Tajima’s *D*, and a comparison of evolutionary rates of HIV and SARS-CoV-2 with the results from NextStrain. Thereon, students were introduced to a diploid restriction associated DNA sequencing (RADseq) dataset from my laboratory, generated from global populations of the invasive Harlequin ladybeetle, *Harmonia axyridis* as part of a population genomics study of global invasive success in the species (Li et al., in review). As part of their laboratory, students utilized plink2 v.2.0.0 [21] for data format conversions, vcftools v.0.1.16 [22] for computing various summary statistics, the qqman [23] package in R for visualization of genome-wide summary statistic estimates, and ADMIXTURE v.1.3.0 [24] for estimation of population structure. The students were then tasked with interpreting the results of these analyses, in congruence with our manuscript.

### Genome-wide association studies

With increased availability of genome-wide data, coupled with large phenotypic data, GWAS are becoming invaluable tools for discovering significantly correlated genomic variants in disease, identifying adaptive loci, and genomic prediction [25]. In a laboratory I designed to introduce students to GWAS, I utilized the best-practices discussed by Marees *et al.* [26] on a genomic–phenotypic dataset from Hayward *et al.* [27] to understand causal variants in congenital deafness in three breeds of dogs. To achieve this, students utilized plink2 [21] for quality control of the data (visualizing population structure using a Principal Components Analysis (PCA), filtering based on minor allele frequencies, missing data content, deviations from Hardy–Weinberg equilibrium, and close relatives) followed by a logistic regression analysis and visualization as Manhattan plots using the qqman [23] package in R. Students were then tasked with assessing congruence of their findings with those reported by the Hayward *et al.* [27] study.

### Differential gene expression analyses, genome annotation

**Figure 1:**
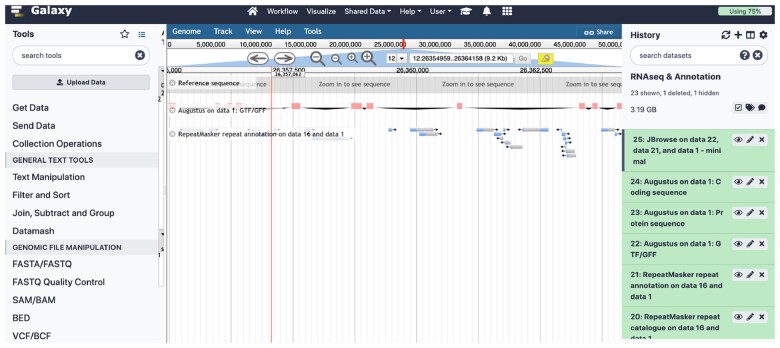
JBrowse instance of a *D. rerio* chromosome 12 annotation produced by AUGUSTUS and RepeatMasker, visualized using the Galaxy Project interface. The full history for these analyses can be accessed at: https://usegalaxy.org/u/arun_sethuraman/h/rnaseq–annotation.

In order to familiarize students to the Galaxy Project (www.usegalaxy.org), another invaluable toolbox for inclusive and accessible genomic data analyses, I incorporated a laboratory on differential gene expression analyses, adapted from Myrto Kostadima’s RNA-seq tutorial from EMBL (bpa-csiro-workshops.github.io), that (i) aligns RNAseq data generated from two developmental stages of zebrafish (*Danio rerio*) to the reference genome using TopHat v.2.1.1 [28], (ii) assembles whole transcriptomes and computation of feature statistics using Cufflinks v.2.2.1 [29], (iii) differential gene expression analyses and multiple-testing correction using CuffDiff v.2.2.1 [29], and (iv) functional enrichment of differentially expressed genes using the DAVID [30, 31] database, all performed in a simple Galaxy Project pipeline. The following laboratory, students performed annotation of the *D. rerio* genome (chromosome 12) using two pipelines—(i) repeat-masking using RepeatMasker v.4.0 [32] against the Dfam v.3.6 [33] database, (ii) *ab initio* gene prediction using AUGUSTUS v.3.4.0 [34], (iii) guided annotation using the MAKER v.2.31.11 [35] pipeline, and (iv) creation of a combined genome browser instance using JBrowse v.1.16.11 [36] (Fig. 1). The lecture portion of these analyses permitted broaching topics related to elevated false discovery rates due to multiple-testing and different correction methods, as well as an introduction to hidden Markov models for genome annotation and the utility of the Viterbi and backward/forward algorithms for inference as implemented in the hmm package in R (https://cran.r-project.org/web/packages/HMM/HMM.pdf).

## Reinforcement of concepts

Recognizing the sheer breadth of concepts and analyses covered during the course of the semester, I wanted to ensure that there was sufficient reinforcement of concepts through regular assignments, review sessions, and take-home examinations which have been shown to increase retention and recapitulation [37]. Here, I describe some of the student exercises, with the entire set of curricular materials available through the course’s GitHub page.

### Programming assignments

Students utilized a programming language and coding environment of their choice (e.g. Python, R, and Java) to (1) develop a tool that took a FASTQ file as input, computed the distribution of PHRED quality scores from the ASCII Illumina encoding (https://support.illumina.com/help/BaseSpace_OLH_009008/Content/Source/Informatics/BS/QualityScoreEncoding_swBS.htm), and produce a visual summary of the per-nucleotide quality distribution, (2) take a reference genome as a FASTA file, and construct the Burrow–Wheeler transform and suffix array encoding of the genome, (3) implement a search algorithm (e.g. backward search algorithm [38], or bisection algorithm [39] for a set of reads provided as a FASTQ file in the reference genome, (4) write a Wright–Fisher simulation of allele frequencies in a diploid, bi-allelic population, and visualize the rates of drift in populations of varying size, and (5) simulate trajectories of genotype frequencies in a small inbred population.

#### Scripting genomic analysis pipelines

Through the semester, students were also tasked with analyses of publicly available genomic datasets including the take-home final examinations, where they submitted a shell script that encompassed all steps in assembly, quality control, trimming, quality assessment, phylogeny reconstruction, annotation, and creation of a genome browser instance from a whole-genome sequencing run of the bacterium *Treponema pallidum* subsp. *pallidum* (SRA Accession: SRR18326765). They also had to perform analyses of population structure in the species using the data of Beale *et al.* [40], and assess congruence of their results with those from the manuscript and discuss the evolutionary history of the species. Other exercises that were assigned to them included tracing the evolutionary origins of the HIV-1 epidemic using data from Faria *et al*. [41] by constructing phylogenetic trees, (2) assembly, annotation, quality control, and analyses of whole genomes from *Escherichia coli* (ENA Accession ID: SRR957824), and reanalyses of population genomic summary statistics and estimates of population structure in a resident song sparrow, *Melospiza melodia* from Mikles *et al.* [42].

### Project reports

Students were also provided with the resources and time on their Jetstream machines to work on an independent project that incorporated techniques, analyses, and concepts learned through the semester. These independent projects spanned a wide range of topics that included temporal phylogenomic analyses of SARS-CoV-2 whole genomes in pre-vaccinated and post-vaccinated time points, GWAS of physiological phenotypes among human populations from high altitude regimes in Tibet, analyses of native Australian population structure in *Eucalyptus moluccana*, and the development of an RNAseq differential gene expression pipeline in the Galaxy Project. Some sample independent project reports from the course are shared with permission from students and their research mentors on the course’s GitHub page.

## Assessment and student evaluations

Students were formally assessed at the end of the semester and provided with several question prompts that quantify (1) effectiveness of the instructor, (2) stimulation of interest in computational genomics, and (3) effectiveness of the evaluative components on a scale of 1–5 (1 = Poor, 2 = Below average, 3 = Average, 4 = Good, and 5 = Excellent). Across all prompts, responses (*N* = 10) indicated that this course, instructional material, and my teaching proved effective (Table 2, mean = 4.81, standard deviation = 0.46, and median = 5.00). None of the enrolled students dropped the course at any point in the semester, and they nearly exhaustively utilized all allocated CPU hours on their respective virtual machines. A majority of the students received an A (95% or more of points), or an A− (90–95% of points), which indicated continued interest in the curricular material throughout the semester.

**Table 2:** Collated responses for 12 quantitative assessment questions on a scale of 1–5 (1 = Poor, 2 = Below average, 3 = Average, 4 = Good, and 5 = Excellent) from *N* = 10 students from Spring 2022

Question prompt	Mean	Standard deviation	Median
Course organization and presentation	5.00	0.00	5.00
Focus on course learning objectives	4.90	0.32	5.00
Overall teaching	4.80	0.42	5.00
Enhancement of understanding of subject	4.50	0.71	5.00
Contribution of examinations to the learning process	4.60	0.52	5.00
Contribution of assignments to the learning process	4.80	0.42	5.00
Effective use of class time	4.80	0.42	5.00
Clarity of presentation of materials	4.60	0.70	5.00
Instructor’s helpfulness	4.90	0.32	5.0
Instructor’s responsiveness	5.00	0.00	5.00
Instructor’s mastery of the subject matter	5.00	0.00	5.00
Instructor’s stimulation of your interest in the subject	4.80	0.63	5.00
Overall	4.81	0.46	5.00

*Notes*: These questions assessed teaching effectiveness, quality of curricular material, and contributions of evaluative components. Overall effectiveness score from Spring 2022 was 4.81 (0.46 standard deviation).

While it was definitely challenging to maintain an engaging environment in a hybrid format, I hope that the convenience of it outweighs the difficulties. I also hope that open-sourcing all my curricular material will encourage more bioinformatics and genomics instructors to adopt this hybrid instructional strategy in the future.

## Conclusions

Teaching applied bioinformatics and computational genomics, especially at the graduate and undergraduate levels is challenging, especially considering that not all institutions have equitable access to HPC to permit large-genomic data analyses in an instructional scenario. I hope that this course modeled on XSEDE’s Jetstream will encourage more instructors to utilize it for instructional purposes on similar education platforms. While this was an ambitious course, I am also hopeful that other instructors will be able to adapt my curricular materials to their own specialties, styles, and pace.

## Data Availability

All curricular materials are accessible via the course’s GitHub page: www.github.com/arunsethuraman/biomi609spring2022.

## Notes

**Arun Sethuraman** is an Assistant Professor in the Department of Biology at San Diego State University. His laboratory develops new statistical methods and software for the estimation of evolutionary history and genetic similarity from large population genomic datasets. More information on his research and teaching can be found on his website: www.arunsethuraman.weebly.com.
